# Relationships between Foot Morphology and Foot Muscle Strength in Healthy Adults

**DOI:** 10.3390/ijerph17041274

**Published:** 2020-02-17

**Authors:** Songlin Xiao, Xini Zhang, Liqin Deng, Shen Zhang, Kedong Cui, Weijie Fu

**Affiliations:** 1 School of Kinesiology, Shanghai University of Sport, Shanghai 200438, China; xiao_songlin@126.com (S.X.); zhangxini1129@163.com (X.Z.); 18873286059@163.com (L.D.); zhangshen0708@163.com (S.Z.); 15618971770@163.com (K.C.); 2 Key Laboratory of Exercise and Health Sciences of Ministry of Education, Shanghai University of Sport, Shanghai 200438, China

**Keywords:** foot morphology, toe flexor strength, metatarsophalangeal joint flexor strength, correlation

## Abstract

The purpose of this study was to investigate if measurements of foot morphology in sitting and standing positions can predict foot muscle strength. Twenty-six healthy male adults were recruited, and their foot morphology and foot muscle strength were measured. Foot morphological variables, toe flexor strength, and metatarsophalangeal joint flexor strength were measured by using a digital caliper, Ailitech-AFG500 dynameter and metatarsophalangeal joint flexor strength tester, respectively. Partial correlation and multivariate stepwise regression were used to explore the relationships between foot morphology and toe/metatarsophalangeal joint strength. Results adjusted by age and body mass index were as follows: (1) truncated foot length in sitting and standing positions and foot width in standing position were positively correlated with the flexor strength of the first toe; (2) foot length, foot width, and truncated foot length in both positions were positively related to the flexor strength of the other toes; (3) arch height index in sitting position and differences in navicular height were negatively associated with the flexor strength of the other toes; (4) differences in foot width were negatively associated with metatarsophalangeal joint flexor strength; and (5) the multivariate stepwise regression model showed that truncated foot length in sitting position, navicular height in standing position, differences in navicular height, foot width in sitting position, and differences in foot width were significantly correlated with toe/metatarsophalangeal joint flexor strength. Simple measurements of foot morphological characteristics can effectively predict foot muscle strength. Preliminary findings provided practical implications for the improvement of the foot ability by making specific foot muscle training sessions in professional sports and by compensating the predicted muscle strength defects to prevent foot injury.

## 1. Introduction

About five million years ago, humans further evolved and developed complex foot morphological characteristics (e.g., heel, arch, and enlarged first metatarsal bone) to adapt to the needs of various activities, such as upright standing, walking, and endurance running [1]. As the direct complex contact area between the lower extremities and the ground/interface, the human foot plays an important role in supporting body mass in both static and dynamic upright locomotion [2,3] and is controlled by intrinsic and extrinsic foot muscles [4]. On account of the complexity of the foot structure, morphological parameters (e.g., foot length, foot width, foot arch height) have been utilized to simplify the anatomical complexities of the foot and describe foot type in the clinical field [5,6].

Current studies have focused on the relationships between foot morphology and foot muscle function. Hillstrom et al. [5] reported that differences in foot morphology are associated with differences in foot function during movements, especially weight-bearing locomotion. Yamauchi et al. [7] revealed that the increase in the force-generating capacity of the foot in upright standing is affected by foot arch dynamics (differences in foot arch height between sitting and standing positions). These studies suggested that variations in human foot morphology are functionally adapted to various motor tasks. Besides, different foot morphologies may have a negative impact on the quality of life in children [8], especially in an adult female with foot problems [9]. Thus, how the functional role of foot morphology is related to foot muscle function needs to be explored to predict foot muscle function, prevent foot injury, and promote health.

Toe flexor muscles comprise the intrinsic and extrinsic foot muscles [10], which are deemed as local stabilizers and global movers, respectively [4]. Toe flexor strength is considered an important factor affecting walking and standing abilities and balance stability [11]. Specifically, the weakness of toe flexor muscles may cause pain and movement problems [12] and increase the risk of falls in elderly individuals [13]. However, efficient strength measurements of these muscles are challenging due to the lack of precise measuring equipment [14]. Feasible measurements of foot morphology are clinically practical and less costly than equipment-based measurements of foot muscles [6], such as MRI and ultrasound [15,16]. Previous studies proved the feasibility of the concept that foot morphology is related to function [6,17]. Uritani et al. [18] used a dynamometer to evaluate toe grip strength and found that toe grip strength is substantially and positively correlated with individual anthropometrics and foot arch height. The relationship between toe deformation and decreased toe flexor strength has been demonstrated by Mickle et al. [13]. Toe flexor strength is also positively correlated with arch height [11,19]. Hence, assessing foot muscle function by measuring foot morphological characteristics, including foot length, foot width, truncated foot length, and navicular height, etc., can result in better understanding of the in-depth relationships between foot muscle strength and foot morphological characteristics and can provide references for athlete selection.

Therefore, the purpose of this study was to investigate if measurements of foot morphology in sitting and standing positions can evaluate or predict foot muscle strength. We hypothesized that foot length, foot width, truncated foot length, and arch height are significantly correlated with toe/metatarsophalangeal joint flexor strength.

## 2. Materials and Methods

### 2.1. Participants

Twenty-six healthy male adults (age: 24.5 ± 3.6 years; height: 176.1 ± 6.6 cm; body mass: 72.4 ± 11.5 kg; body mass index [BMI]: 23.3 ± 2.87 kg/m^2^) were recruited. The participants regularly participated in aerobic activities. Inclusion criteria included having no musculoskeletal injuries of the lower extremities over the previous six months, the right leg was the dominant lower extremity, and both feet must be rectus foot type. All participants were provided an informed consent approved by the Institutional Review Board of the Shanghai University of Sport before the tests (IRB no. 2017007).

### 2.2. Data Collection

The height, weight, and age of the participants were recorded before the tests. All procedures and potential hazards were explained to the participants in nontechnical terms to ensure they were fully knowledgeable of test procedures and requirements. All participants signed the relevant questionnaire and informed consent. The participants performed a 5 min warm-up on a treadmill, followed by formal foot morphology measurements and foot muscle strength assessment by the same physical therapist.

#### 2.2.1. Foot Morphology in Standing and Sitting Positions

**Figure 1:**
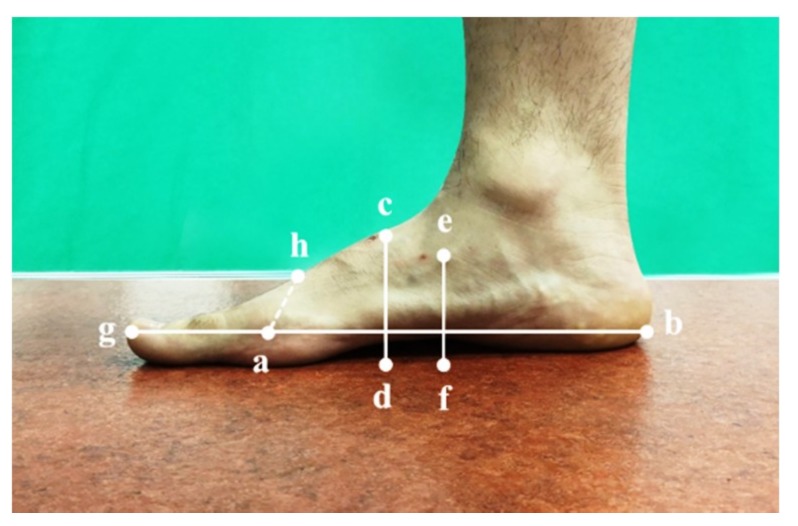
Schematic of foot morphological measurement. L_gb_: foot length; L_ah_: foot width; L_ab_: truncated foot length; L_ef_: navicular height; L_cd_: dorsum height at 50% of the foot length.

Foot morphology in the standing position was assessed by requiring the participants to stand on the measuring platform barefoot, with both feet parallel to each other and about hip-width apart, toes forward, hands placed naturally down, head faced forward, and trunk without flexion or rotation. Each foot bore approximately 50% body weight of loading in a standing position [20]. All foot morphological variables were measured using a digital caliper (S-Cal Pro910.1502, Sylvac, Yverdon, Switzerland) [21] (Figure 1).

Foot morphology in the sitting position was assessed by asking the participants to sit on a seat with adjustable height. Their thighs were parallel to the floor, their knee remained in flexion of 90°, and the remaining requirements were the same as described in standing position. Each foot sustained no weight-bearing in a sitting position [20]. All morphological tests were performed by the same physical therapist.

#### 2.2.2. Toe Flexor Strength

**Figure 2:**
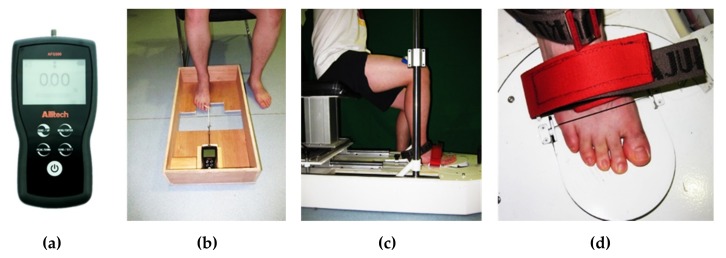
(**a**) Digital display dynamometer; (**b**) The experimental setup of toe flexor strength of measurement; (**c**,**d**) Metatarsophalangeal joint flexor strength tester, and the experimental setup.

Toe flexor strength was assessed using a digital dynamometer (Ailitech-AFG500, Ailitech, Guangzhou, China) (Figure 2a). This method has excellent reliability and reproducibility [14]. The participants were asked to sit on a chair with their knee and ankle joints adjusted to 90° flexion, and their calcaneus kept against the frame of the nearest set of board. The board under the foot provided support from the heel to the head of the first metatarsal, thereby allowing toe flexion. Hallux flexion strength was tested by tying the first toe with rigid toe rings wrapped with sponge, which was connected to the head of the digital display dynameter by a rigid connecting rod. The digital display dynameter was installed and fixed at the opposite frame of the wooden case. Mainly contracting abductor hallucis and flexor hallucis brevis, the participants flexed the first toe and pulled the ring back as hard as possible (Figure 2b). Then, the toe ring was replaced to fit the size of the other four toes (2nd to 5th toes) to measure the flexor strength of the other toes following the same procedure as described above, and the special muscles being tested mainly included flexor digitorum brevis, abductor digiti minimi, lumbricals, and flexor digiti minimi. Three trials were performed, followed by a one-minute break after each trial.

#### 2.2.3. Metatarsophalangeal Joint Flexor Strength

Metatarsophalangeal joint flexor strength was measured using a customized metatarsophalangeal joint flexor strength tester (Patent No. CN103278278A, China) with a sampling frequency of 120 Hz (Figure 2c). Details of the tester and its testing process are provided in our previous study [22]. Briefly, the participants sat on a chair with their knee and feet fixed by a stopper plate to avoid the interference of other joints. During the tests, the participants were required to try their best to flex all of their plantar muscles together for 10 s to press the 30° raised pedal (Figure 2d). Measurements were repeated three times, with a one-minute break between trials.

### 2.3. Data Processing

All data were recorded and collated by the same physical therapist. The measured foot morphological variables included foot length, truncated foot length, foot width, navicular height, and dorsum height at 50% of the foot length. The definition of the above variables is presented in Table 1. Additionally, the arch height index (AHI) was calculated as the dorsum height at 50% of the total foot length divided by truncated foot length, according to Williams et al. [21] (Table 1). Peaks of the flexor strength of the first toe, the other toes, and metatarsophalangeal joints were normalized by body weight. The average values of the three trials were extracted.

### 2.4. Statistics

Data are presented as mean ± standard deviation. All dependent variables were normally distributed, as indicated by the Shapiro–Wilk test. Comparisons of foot morphology in sitting and standing positions were performed using paired *t*-tests. Pearson correlation and Partial correlation were used to calculate the correlation between foot morphological variables and toe/metatarsophalangeal joint muscle strength with and without adjusted age and BMI. Correlation coefficient *r* was reported to Pearson correlation results without adjusted age and BMI. Correlation coefficient *r* (adjusted) was reported to Partial correlation results with adjusted age and BMI. Multivariate analysis by stepwise regression was utilized to model the correlation of foot morphological variables and toe/metatarsophalangeal joint muscle strength (SPSS 22.0, SPSS Inc., Chicago, IL, USA). The significance level was set as α = 0.05. Correlation coefficient *r*: |*r*| ≥ 0.8 indicates high correlation; 0.5 ≤ |*r*| < 0.8 denotes moderate correlation; 0.3 ≤ |*r*| < 0.5 suggests low correlation; and |*r*| < 0.3 implies weak correlation.

## 3. Results

### 3.1. Foot Morphology and Foot Muscle Strength

Foot length and width increased significantly in standing position compared with in sitting position (*p* < 0.001) (Table 2). Navicular height, dorsum height at 50% of the foot length, and AHI significantly decreased in standing position compared with in sitting position (*p* < 0.001). Foot muscle strengths of all participants were as follows: the flexor strength of the first toe, of the other toes, and of the metatarsophalangeal joint was 1.54 ± 0.44, 0.95 ± 0.29, and 1.35 ± 0.51 N/kg, respectively.

### 3.2. Relationships Between Foot Morphology and Toe Flexor Strength

**Figure 3:**
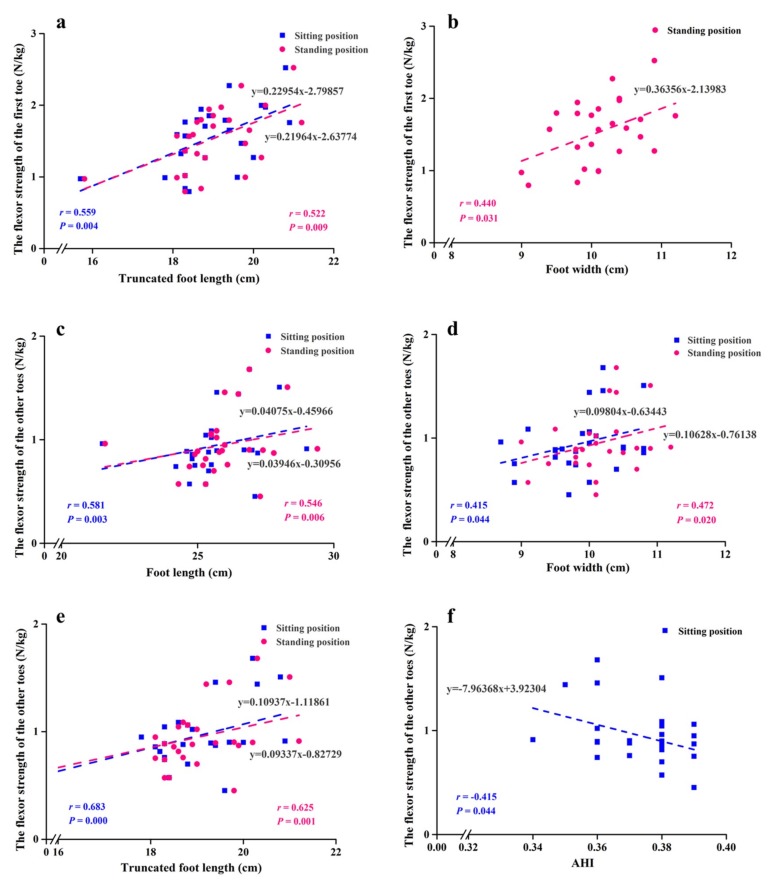
Correlations of foot morphology with toe flexor strength adjusted to age and BMI. (**a**) Relationship between the flexor strength of the first toe and truncated foot length in sitting and standing positions. (**b**) Relationship between the flexor strength of the first toe and foot width in standing position. (**c**–**e**) Relationships between the flexor strength of the other toes and foot length, foot width, and truncated foot length, respectively, in sitting and standing positions. (**f**) Relationship between the flexor strength of the other toes and AHI in sitting position.

The flexor strength of the first toe unadjusted to age and BMI was positively correlated with foot width and truncated foot length (*r* = 0.427–0.561, *p* < 0.05) in sitting and standing positions and with foot length (*r* = 0.390, *p* = 0.049) and dorsum height at 50% of the foot length (*r* = 0.407, *p* = 0.039) in sitting position (Table 3). The flexor strength of the first toe adjusted to age and BMI was positively correlated with truncated foot length in sitting and standing positions (*r* = 0.559, *p* = 0.004; *r* = 0.522, *p* = 0.009, respectively) (Figure 3a) and with foot width in standing position (*r* = 0.440, *p* = 0.031) (Figure 3b). The flexor strength of the other toes was positively correlated with truncated foot length (*r* = 0.403, *p* = 0.041) in sitting position (Table 3). The flexor strength of the other toes adjusted to age and BMI was positively correlated with foot length, foot width and truncated foot length (*r* = 0.415–0.683, *p* < 0.05) (Figure 3c,e) and negatively correlated with AHI (*r* = −0.415, *p* = 0.044) in sitting position (Figure 3f). 

### 3.3. Relationships Between Foot Morphology and Metatarsophalangeal Joint Flexor Strength

**Figure 4:**
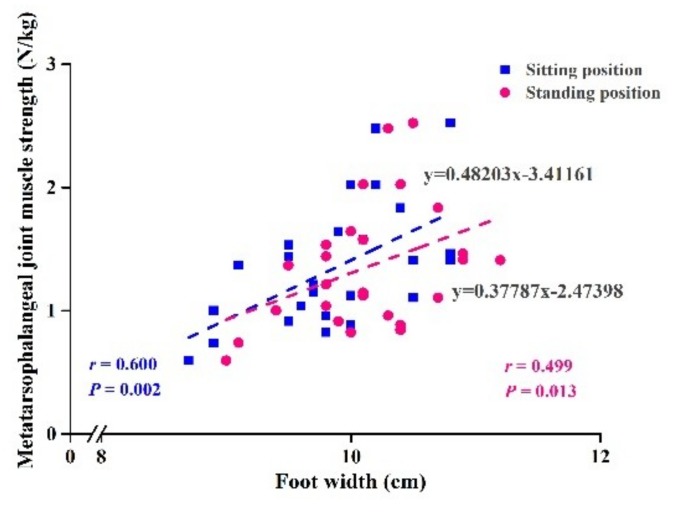
Correlations between foot width and metatarsophalangeal joint flexor strength adjusted to age and BMI in sitting and standing positions.

Metatarsophalangeal joint flexor strength was positively correlated with foot width in sitting and standing positions with and without adjustments for age and BMI (*r* = 0.399–0.600, *p* < 0.05) (Table 4). The linear relationships are shown in Figure 4.

### 3.4. Relationships Between the Differences in Foot Morphology and Foot Muscle Strength

The flexor strength of the other toes adjusted to age and BMI was negatively correlated with differences in navicular height (*r* = −0.425, *p* < 0.05). Metatarsophalangeal joint flexor strength adjusted to age and BMI was negatively correlated with differences in foot width (*r* = −0.515, *p* < 0.01) (Table 5).

### 3.5. Regression Model of Relationships Between Foot Morphology and Foot Muscle Strength

The regression model indicated that (1) the flexor strength of the first toe was correlated with truncated foot length in sitting position, (2) the flexor strength of the other toes was associated with truncated foot length in sitting position, navicular height in standing position and differences in navicular height, and (3) metatarsophalangeal joint flexor strength was correlated with foot width in sitting position and differences in foot width (Table 6).

## 4. Discussion

In this study, foot morphological characteristics and foot muscle strength were measured to explore the relationship between foot morphology in standing and sitting positions and toe/metatarsophalangeal joint flexor strength. The major findings of this study demonstrated that foot length, foot width, and truncated foot length adjusted to age and BMI were positively correlated to toe/metatarsophalangeal joint flexor strength. AHI and differences in navicular height were negatively associated with the flexor strength of the other toes. Finally, the multivariate stepwise regression model showed that truncated foot length, navicular height, differences in navicular height, and differences in foot width were substantially correlated with toe/metatarsophalangeal joint flexor strength. This study explained the correlations between foot morphology and foot muscle strength in detail, and the hypothesis was accepted.

### 4.1. Foot Morphology in Sitting and Standing Positions

Individuals commonly maintain a sitting or standing position. Different load bearings under the two positions would lead to obvious foot morphological characteristics. However, previous studies paid little attention to the distinction of foot morphology under the two positions. This study compared the variables of foot morphology under the two positions. Results showed that foot length and foot width remarkably increased, whereas navicular height, dorsum height at 50% of the foot length, and AHI considerably decreased in standing position compared with in sitting position. This consequence could be attributed to the structural deformation caused by the foot skeleton supporting the weight. The foot bears a little body weight in the sitting position, whereas the whole body weight transfers to the foot in the standing position with the calcaneus acting a supporting role and flattening the medial longitudinal and transverse bows [20]. Especially, the decrease in the height of the medial longitudinal arch resulted in a corresponding increase in foot length, whereas the lower height of the transverse arch generated a wider foot width in this study.

### 4.2. Relationships between Foot Morphology and Foot Muscle Strength

Foot length and foot width can predict the height and shape of an individual, characteristics that are critical to identifying individuals in criminal investigations [26], war, and disaster [27]. However, whether foot morphology could predict foot muscle strength is unclear. To the best of our knowledge, few studies have focused on investigating the relationships between foot morphology and ankle muscle strength. Zhao et al. [28] collected foot morphology and ankle muscle strength through a foot scanner and dynamometer, respectively, and found that foot length and foot width are positively correlated with ankle joint muscle strength. Our study showed that foot length, foot width, and truncated foot length were correlated with toe flexor strength through similar methods of foot morphology measurements. Moreover, foot width was moderately correlated with metatarsophalangeal joint flexor strength. The results of this study suggested that participants with longer foot length and width may have a longer force arm during toe flexion, which increases the moment of foot flexion and generates greater force. On the other hand, muscles can be sufficiently stretched before muscle shortening to increase the force production of the muscle due to longer foot length and width [29]. 

Moreover, AHI in sitting position was negatively associated with the flexor strength of the other toes, that is, flexor strength increased with decreasing arch height. Foot arch plays a spring-like role during movements [30]. People with lower arches tend to have more flexible feet, whereas those with higher arches are more likely to have stiffer feet [31]. Lower arches could flexibly absorb more ground reaction force during activities than high arches. Lower arches require more effort to control the stability of foot structures and maintain body balance, which needs great foot muscle strength to cope with the increased ground reaction force during walking and running [32]. Our findings support this viewpoint. Although a previous study deemed that judging whether lower arches are a sign of physiological adaptations or pathological changes is difficult [33], the results of our study showed that a lower arch height is more likely a physiological adaptation than a pathological change. It was also reported that specific foot and general health-related quality of life did not seem to be influenced by the foot arch height [34]. Different foot types existed, and they affected obvious motor performance in adults, but may not in children [35]. However, several studies also reported that arch height ratio is weakly or even not correlated with toe flexor strength [19,36]. These results differ because the other studies did not consider body position. Foot morphology is different in sitting and standing positions. Furthermore, not separating the first toe from the other toes might be another factor that affected the experimental results of the previous studies. A substantial discrepancy between the flexor strength of the first toe and the remaining four toes was documented [37]. Besides, the relationships between foot morphology and foot muscle strength could be impacted by different foot types. It has been pointed out that planus, rectus, and cavus feet exhibited significantly different measures of foot structure and function [5]. Therefore, more confounding factors, e.g., foot types, postures, and body mass, should be taken into account to further explore the relationship between foot morphology and strength.

### 4.3. Relationships between the Differences in Foot Morphology and Foot Muscle Strength

Differences in navicular height were negatively associated with the flexor strength of the other toes. Furthermore, differences in foot width were negatively correlated with metatarsophalangeal joint flexor strength. This result meant that small differences in navicular height and foot width indicate a great force-generating capacity of metatarsophalangeal joint and toes, respectively. Moreover, the results of the multivariate stepwise regression model showed that differences in navicular height and differences in foot width had a significant relationship with foot muscle strength. These preliminary findings indicated that differences in navicular height and differences in foot width may more sensitively assess foot muscle strength. Foot morphology is greatly affected by body weight, and changes in foot morphological characteristics have remarkable differences under different load conditions [38]. Foot length and foot width increase, arch height decrease, and the foot rotates to the medial side (valgus) when foot loads increase from no body weight to partial body weight and then to whole body weight [39]. Compared with in sitting position, the changes in foot morphology induced by body weight-bearing when standing may also affect the length–tension relationship of foot muscles [7], and increasing the vertical load on the foot increases the activities of its intrinsic muscles [40]. In addition, the excessive subtalar pronation may limit the dorsiflexion of the hallux and influence the flexion produced by the tension of the plantar fascia [41]. Foot morphology experiences a larger deformation as individuals switch from sitting to standing position [42]. The increase in the force-generating capacity of the foot in upright standing is affected by foot arch dynamics (differences in arch height between sitting and standing positions) [7]. Therefore, a strong foot muscle strength indicates a small difference in foot morphology.

### 4.4. Clinical Applications

It was reported that foot structure and function were related in asymptomatic healthy individuals [6]. Current findings also indicated that foot morphology measurement may predict foot function. Based on mechanically complex structures of the foot, the measures of foot morphology were less costly than foot function and more feasible to be performed in the clinical setting. Besides, these preliminary findings provide practical implications for the improvement of foot ability in making specific foot muscle training sessions and preventing foot injury by predicting weak muscle strength.

### 4.5. Limitations

Several limitations inherently existed in this study. On the one hand, the sample size was small and confounding factors, such as physical activity level, age, height, and gender, were not considered. These factors may have influenced the measurement of several foot morphological variables. Future studies are needed to evaluate the effect of these confounding factors on predicting foot function by using the logistic regression model and stepwise linear regression. On the other hand, foot morphology is positively related to body mass. Therefore, we need to control or stratify variables further and investigate the relationships between foot morphology and foot muscle strength in specific populations, such as in overweight and obese sedentary participants in a future study.

## 5. Conclusions

Foot length, foot width, and truncated foot length adjusted to age and BMI were positively correlated with toe/metatarsophalangeal joint flexor strength. AHI in sitting position, differences in navicular height, and differences in foot width were negatively correlated with toe/metatarsophalangeal joint muscle strength. Multiple linear relationships suggested that measuring foot morphological characteristics can effectively predict foot muscle strength. These preliminary findings provide practical implications for the improvement of foot ability in making specific foot muscle training sessions in professional sports, such as running and jumping events, and by compensating the predicted muscle strength defects to prevent foot injury.

## Figures and Tables

**Table 1:** Measured foot morphological variables.

Variables	Definition	Source
Foot length (cm)	Measured from the most posterior point of the calcaneus to the distal end of the longest toe	Butler et al., 2008 [23]
Foot width (cm)	Measured from the first metatarsophalangeal joint to the fifth metatarsophalangeal joint	Zhao et al., 2017 [24]
Truncated foot length (cm)	Measured from the most posterior point of the calcaneus to the first metatarsophalangeal joint	Butler et al., 2008 [23]
Navicular height (cm)	Measured from the ground to the tuberosity of navicular bone	Mulligan et al., 2013 [25]
Dorsum height at 50% of the foot length (cm)	Obtained at 50% of foot length with a vertical caliper measured from the ground to the top of the dorsum	Mulligan et al., 2013 [25]
Arch height index (AHI)	Calculated as the dorsum height at 50% of the total foot length divided by truncated foot length	Williams et al., 2000 [21]

**Table 2:** Foot morphological characteristics of all participants.

Variables	Sitting Position	Standing Position	Effect Size	Diff.
Foot length (cm)	25.71 ± 1.42	25.95 ± 1.47 **	1.09	0.23 ± 0.22
Foot width (cm)	9.88 ± 0.58	10.12 ± 0.54 **	1.21	0.24 ± 0.20
Navicular height (cm)	5.44 ± 0.45	4.91 ± 0.54 **	1.15	0.53 ± 0.46
Dorsum height at 50% of the foot length (cm)	7.03 ± 0.38	6.53 ± 0.41 **	2.46	0.50 ± 0.20
Truncated foot length (cm)	18.90 ± 1.08	19.02 ± 1.08	0.4	0.12 ± 0.30
AHI	0.37 ± 0.01	0.34 ± 0.02 **	1.95	0.03 ± 0.01

** A significant difference between sitting and standing position at *p* < 0.01. Effect size values were reported to paired *t*-tests results. Diff.: Difference in relative foot morphological characteristics between sitting and standing positions.

**Table 3:** Correlation coefficient of foot morphology and toe flexor strength.

Toe Flexor Strength	Position	Foot Morphology	*r*	*p*-Value	*r* (Adjusted)	*p*-Value
Flexor strength of the first toe	Sitting position	Foot length	0.390	**0.049**	0.327	0.118
		Foot width	0.427	**0.029**	0.393	0.057
		Navicular height	−0.003	0.990	−0.208	0.330
		Dorsum height at 50% of the foot length	0.407	**0.039**	0.324	0.122
		Truncated foot length	0.561	**0.003**	0.559	**0.004**
		AHI	−0.341	0.088	−0.362	0.083
	Standing position	Foot length	0.373	0.061	0.299	0.155
		Foot width	0.440	**0.025**	0.440	**0.031**
		Navicular height	0.224	0.271	0.137	0.522
		Dorsum height at 50% of the foot length	0.382	0.054	0.295	0.161
		Truncated foot length	0.536	**0.005**	0.522	**0.009**
		AHI	−0.151	0.462	−0.143	0.506
Flexor strength of the other toes	Sitting position	Foot length	0.265	0.191	0.581	**0.003**
		Foot width	0.316	0.115	0.415	**0.044**
		Navicular height	−0.306	0.129	−0.279	0.186
		Dorsum height at 50% of the foot length	0.181	0.377	0.341	0.103
		Truncated foot length	0.403	**0.041**	0.683	**0.000**
		AHI	−0.368	0.065	−0.415	**0.044**
	Standing position	Foot length	0.243	0.231	0.546	**0.006**
		Foot width	0.309	0.125	0.472	**0.020**
		Navicular height	0.131	0.525	0.167	0.434
		Dorsum height at 50% of the foot length	0.174	0.395	0.294	0.164
		Truncated foot length	0.344	0.085	0.625	**0.001**
		AHI	−0.157	0.443	−0.186	0.383

Note: The bold *p*-value means *p* < 0.05.

**Table 4:** Correlation coefficient of foot morphology and metatarsophalangeal joint flexor strength.

Position	Foot Morphology	*r*	*p*-Value	*r* (Adjusted)	*p*-Value
Sitting position	Foot length	0.141	0.491	0.091	0.673
	Foot width	0.550	**0.004**	0.600	**0.002**
	Navicular height	0.065	0.753	−0.028	0.898
	Dorsum height at 50% of the foot length	0.144	0.481	0.071	0.741
	Truncated foot length	0.194	0.342	0.169	0.429
	AHI	−0.090	0.662	−0.130	0.544
Standing position	Foot length	0.110	0.593	0.039	0.855
	Foot width	0.399	**0.044**	0.499	**0.013**
	Navicular height	0.135	0.510	0.054	0.801
	Dorsum height at 50% of the foot length	0.304	0.131	0.275	0.194
	Truncated foot length	0.298	0.139	0.304	0.148
	AHI	0.027	0.897	0.020	0.925

Note: The bold *p*-value means *p* < 0.05.

**Table 5:** Correlation coefficient of differences in foot morphology and foot muscle strength with adjustment to age and BMI.

Variables	Differences in Foot Length	Differences in Foot Width	Differences in Navicular Height	Differences in Dorsum Height	Differences in Truncated Foot Length	Differences in AHI
Flexor strength of the first toe	−0.068	−0.051	−0.332	−0.024	−0.156	−0.186
Flexor strength of the other toes	−0.052	−0.033	−0.425 *	0.003	−0.221	−0.184
Metatarsophalangeal joint flexor strength	−0.212	−0.515 **	−0.086	−0.367	0.342	−0.160

*: A significant correlation at *p* < 0.05; **: A significant correlation at *p* < 0.01.

**Table 6:** Multivariate stepwise regression model of relationships between foot morphology and foot muscle strength.

	Variables	B	SE	β	t	*p*
Flexor strength of the first toe	Constant	−2.799	1.311			
	Truncated foot length in sitting	0.230	0.069	0.561	3.316	0.003
Flexor strength of the other toes	Constant	−0.274	0.810			
	Differences in navicular height	−0.480	0.125	−0.756	−3.853	0.001
	Truncated foot length in sitting	0.155	0.046	0.573	3.405	0.003
	Navicular height in standing	−0.298	0.115	−0.551	−2.598	0.016
Metatarsophalangeal joint flexor strength	Constant	−1.937	1.530			
	Foot width in sitting	0.356	0.150	0.407	2.371	0.026
	Differences in foot width	−0.955	0.439	−0.373	−2.175	0.040

Note: B: unstandardized coefficients. SE: standard error. β: standardized coefficients.
